# Supplementary figures and images for: An intuitive sampling framework for setting-specific decision-making in soil-transmitted helminthiasis control programs
Source: PLoS Negl Trop Dis. 2026 Jun 5;20(6):e0014026. doi: 10.1371/journal.pntd.0014026 (PMC13258144; doi:10.1371/journal.pntd.0014026)

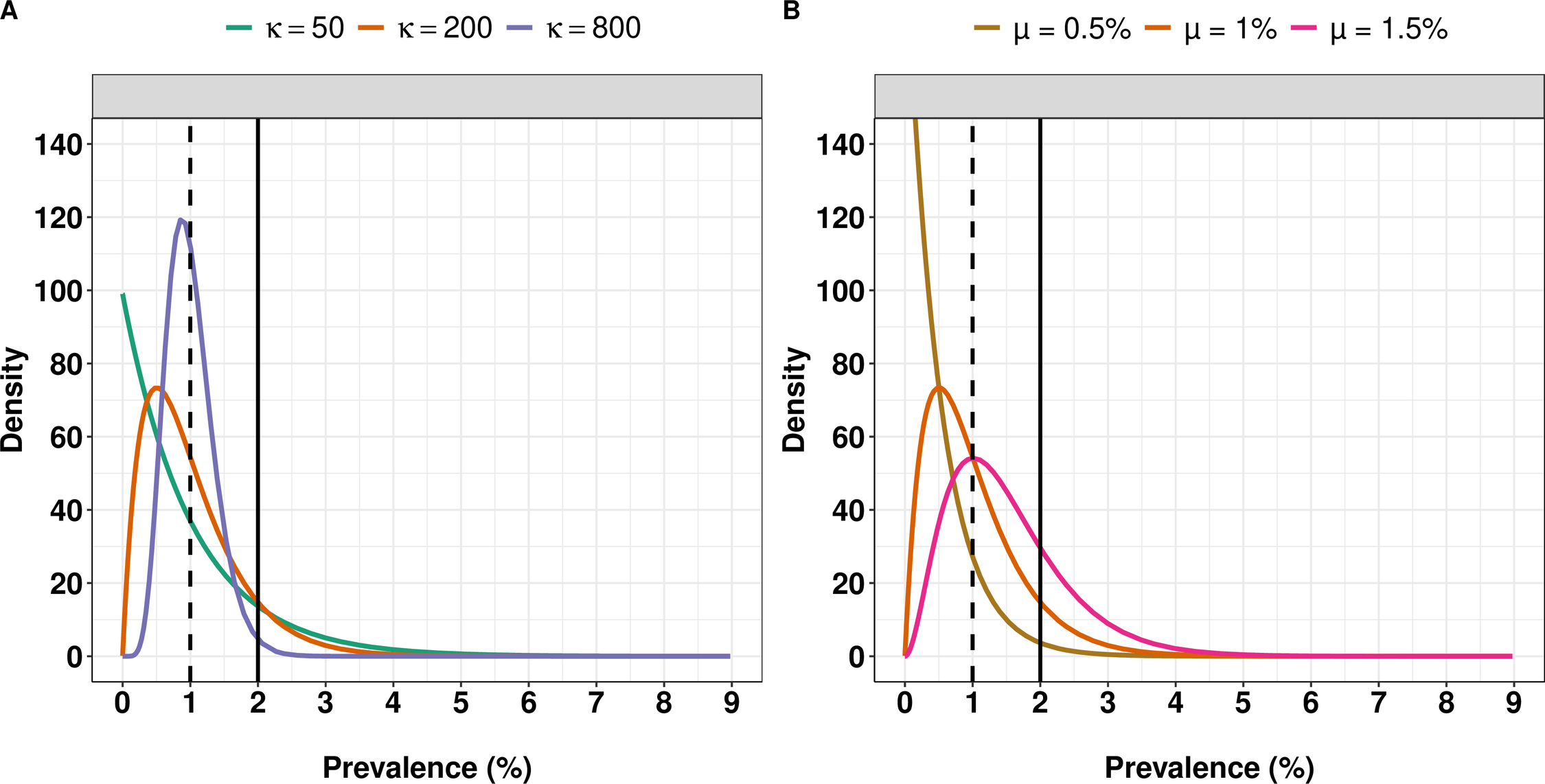

Supplement: S1 Fig — To do this, we first set the mean μ to 1% in Panel A while varying the degree of certainty κ to 50, 200 and 800. In Panel B, we set the mean of degree of certainty to 200, and the mean μ values of 0.5%, 1% and 1.5%. Vertical dotted and solid lines indicate the prior mean of 1% and the prevalence threshold of 2%, respectively. (TIF) [file pntd.0014026.s001.tif]

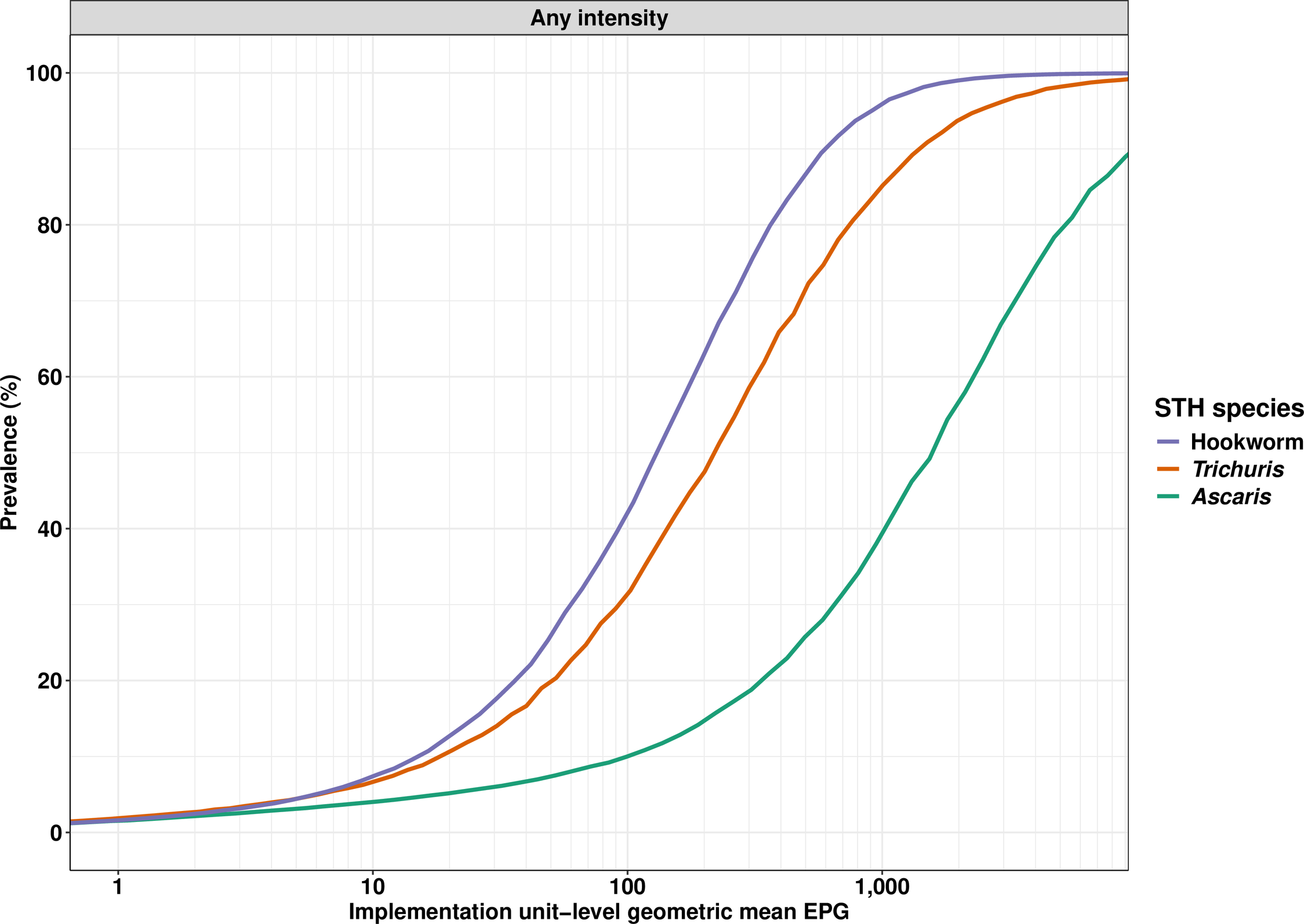

Supplement: S2 Fig — For this, we first determined true prevalence at the implementation unit level when testing 3,000 schools and recruiting 6,000 per school for every mean number of eggs per gram of stool (EPG) when deploying single Kato-Katz thick smear on a single stool sample, accounting for the different sources of variability in egg counts (S1 Table). Note that the logarithm of these mean EPGs was used in our simulation framework. (TIF) [file pntd.0014026.s002.tif]

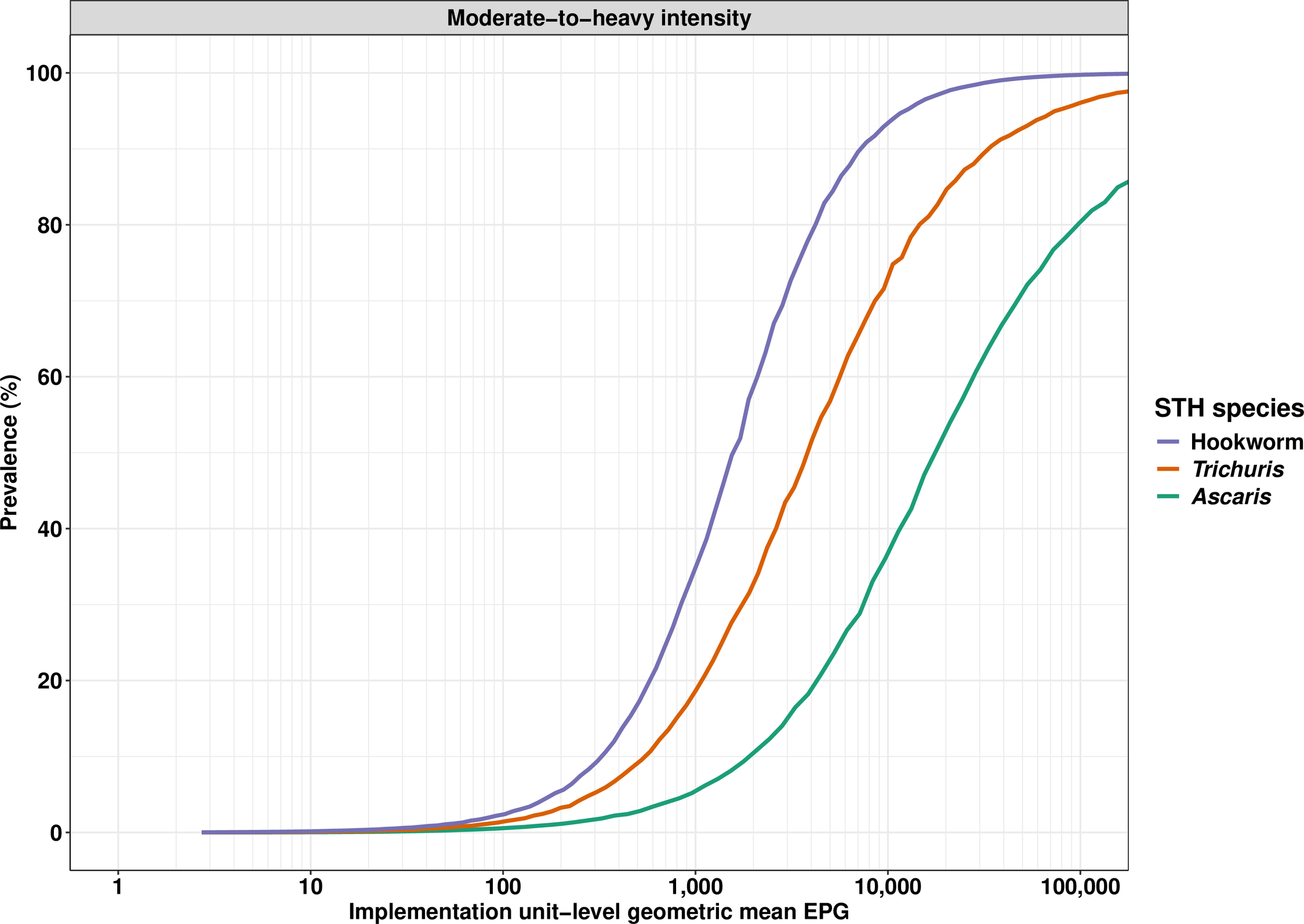

Supplement: S3 Fig — To do this, we first determined true prevalence at the implementation unit level when testing 3,000 schools and recruiting 6,000 children per school for every mean number of eggs per gram of stool (EPG) when deploying single Kato-Katz thick smear on a single stool sample, accounting for the different sources of variability in egg counts (S1 Table). We used the WHO classification of moderate-to-heavy intensity infections (MHI), which is 5,000 EPG for Ascaris, 2,000 EPG for hookworm, and 1,000 EPG for Trichuris. In our simulation, the logarithm of these mean EPG was used. (TIF) [file pntd.0014026.s003.tif]

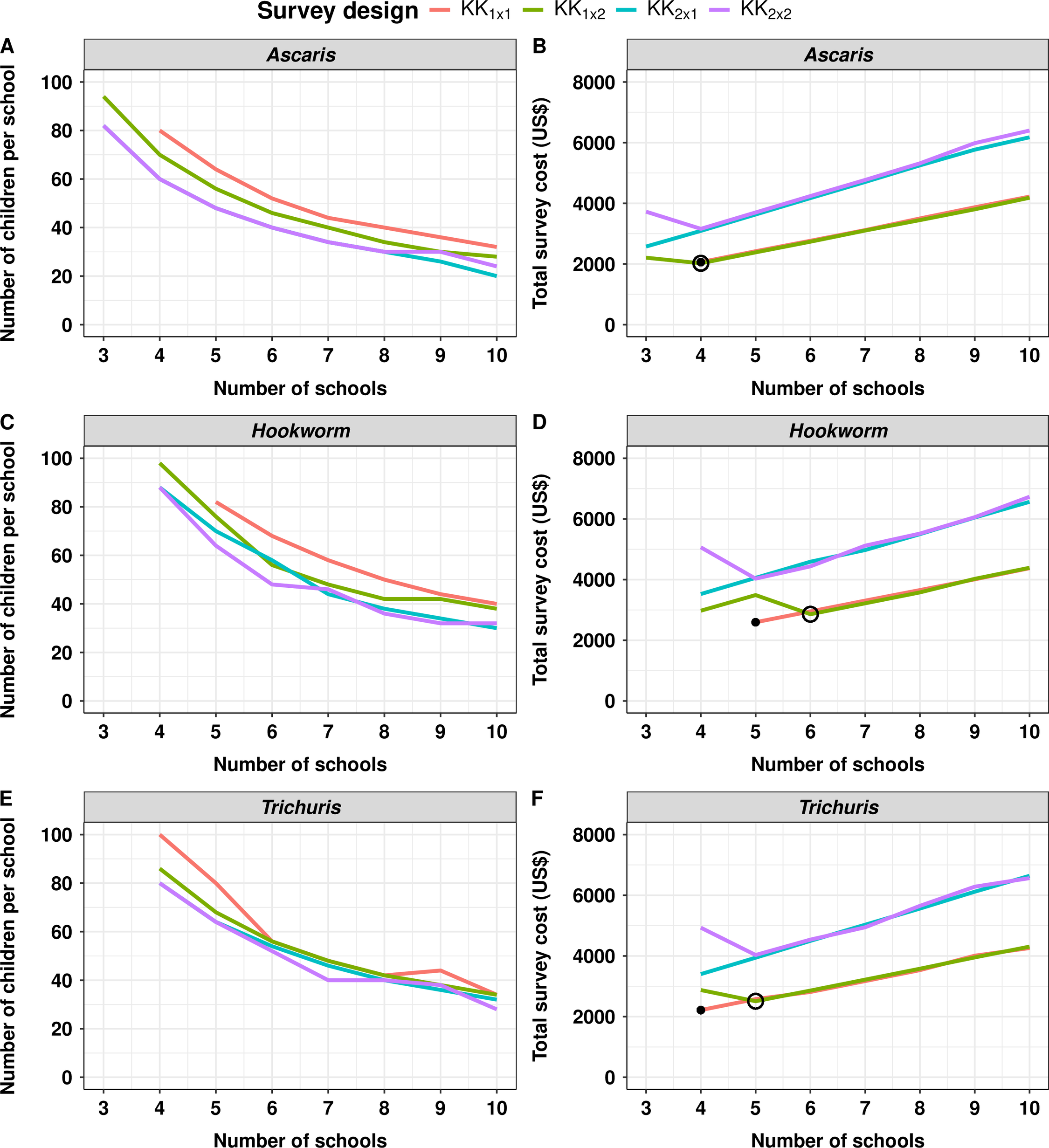

Supplement: S4 Fig — This figure illustrates the required number of children per school (k; Panels A, C and E) and the corresponding total survey cost (μk; Panels B, D and F) as a function of the number of sampled schools (k) for different survey designs and soil-transmitted helminths species (Ascaris: Panels A and B; hookworm: Panels C and D; Trichuris: Panels E and F). The survey designs (β1) varied in the number of stool samples per child (=a) and the number of Kato-Katz thick smears per sample (=b). The black bullet point in Panels B, D and F indicates the most cost-efficient survey design. In other words, the number of schools that minimizes the costs while ensuring reliable decision-making. The open circle indicates the most cost-efficient survey design when preparing two slides per sample, which automatically adds free quality control to the survey design. (TIF) [file pntd.0014026.s004.tif]

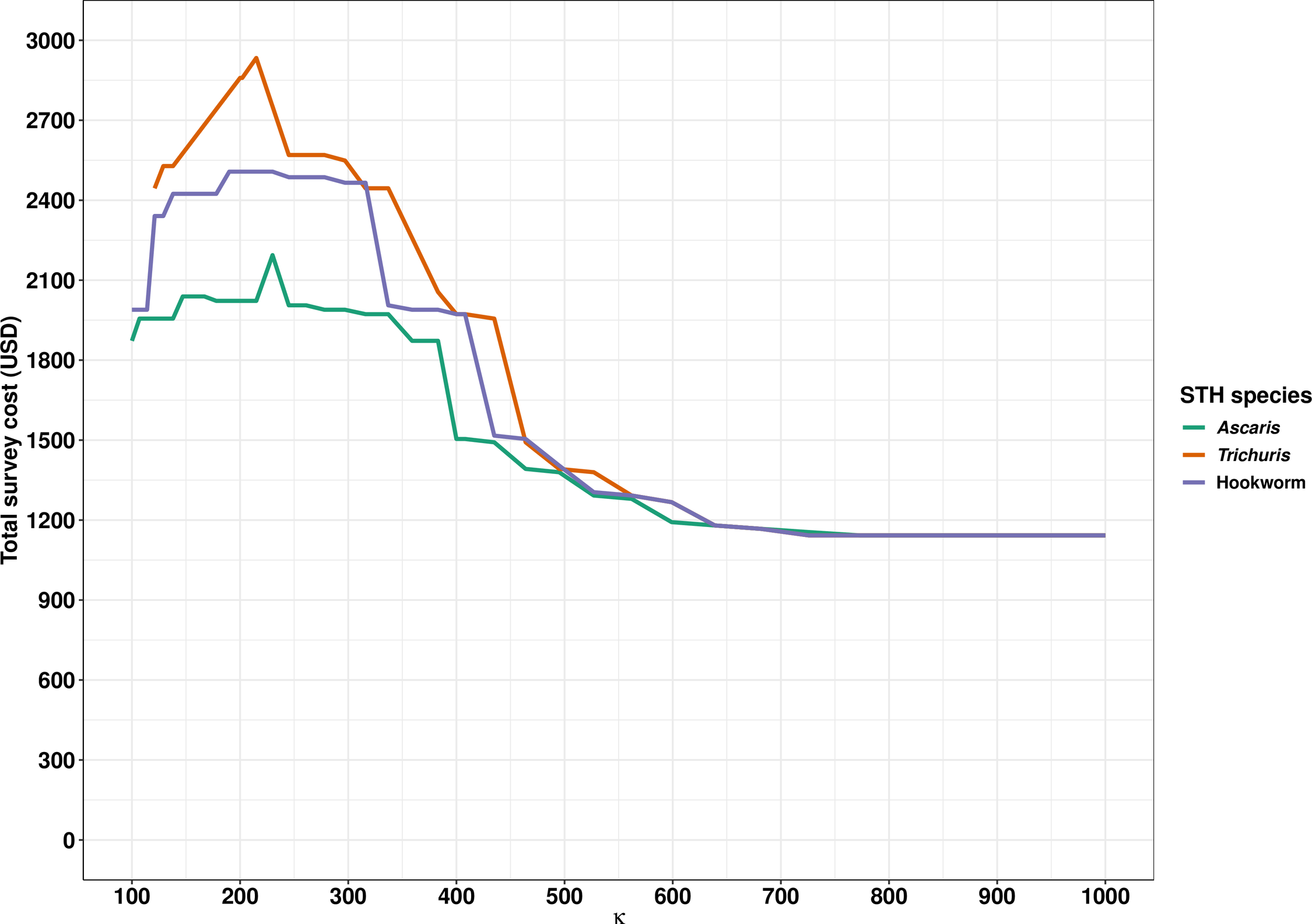

Supplement: S5 Fig — This figure presents the required total survey cost for switching to an event-based PC for each STH species as a function of the degree of certainty (κ). For this, we set the prior mean to 1% and the risk of incorrect decisions to 20% for overtreating and 1% for the risk of undertreating. (TIF) [file pntd.0014026.s005.tif]
