# Supplementary material for: An intuitive sampling framework for setting-specific decision-making in soil-transmitted helminthiasis control programs
Source: PLoS Negl Trop Dis. 2026 Jun 5;20(6):e0014026. doi: 10.1371/journal.pntd.0014026 (PMC13258144; doi:10.1371/journal.pntd.0014026)
Supplement: S1 Table — We parametrized the lognormal distribution of the variability in mean eggs per gram of stool (EPG) across schools using the mean and standard deviation on the logarithmic scale. For the gamma distribution of the inter-individual variability and day-to-day variability in mean EPG, we used the shape parameter nchildren and scale (Ctot), where μ is the distribution’s mean. Note that lower values of nschools mean more heterogeneity and KKa × b was divided by 24 to correct for the scale difference, as the school-level mean is expressed in EPG. (DOCX) [file pntd.0014026.s006.docx]

**Table S1. Parametrisation of the simulation framework for various sources of variability in Kato-Katz thick smear egg counts.** We parametrized the lognormal distribution of the variability in mean eggs per gram of stool (EPG) across schools using the mean and standard deviation on the logarithmic scale. For the gamma distribution of the inter-individual variability and day-to-day variability in mean EPG, we used the shape parameter $k$ and scale ($\frac{\mu}{k}$), where $\mu$ is the distribution's mean. Note that lower values of $k$ mean more heterogeneity and $\beta_{1}$ was divided by 24 to correct for the scale difference, as the school-level mean is expressed in EPG.

| **Parameters** | **STH species** | | | **Data source** |
| --- | --- | --- | --- | --- |
|  | *Ascaris* | Hookworm | *Trichuris* |  |
| Variability in mean EPG across schools within the same district ($\sigma_{i}$) | 0.69 | 0.80 | 0.52 | [12] |
|  |  |  |  |  |
| Intercept ($\beta_{0})$ for school-level aggregation parameter ($k_{k})$ as a linear function of the school-level mean in EPG) | 0.0158 | 0.0162 | 0.0098 | [12] |
|  |  |  |  |  |
| Slope ($\beta_{1}$) for school-level aggregation parameter ($k_{k}$) as a linear function of the school-level mean EPG | 8.04×10^-5^ | 9.28×10^-4^ | 1.85×10^-3^ | [12] |
|  |  |  |  |  |
| Day-to-day variation in EPG within an individual (shape $k_{d}$) | 0.51 | 0.87 | 1.42 | [13] |
