# Supplementary material for: An intuitive sampling framework for setting-specific decision-making in soil-transmitted helminthiasis control programs
Source: PLoS Negl Trop Dis. 2026 Jun 5;20(6):e0014026. doi: 10.1371/journal.pntd.0014026 (PMC13258144; doi:10.1371/journal.pntd.0014026)
Supplement: S2 Table — (DOCX) [file pntd.0014026.s007.docx]

**Table S2. Overview of the cost parameters when deploying KK testing in an Ethiopian setting** [11]

| **Variables** | **Description** | **Cost (US$)** |
| --- | --- | --- |
| **Cost of consumables** | | |
| $cost_{sample}$ | Cost of consumables to collect a single stool sample | 0.57 |
| ${cost}_{aliquot,b}$ | Cost of materials for a single aliquot from the same stool sample | 1.37 |
|  | Cost of materials for a duplicate aliquot from the same stool sample | 0.75 |
|  | | |
| **Personal cost** | | |
| $cost_{salary}$ | Daily salary | 22.50 |
|  |  |  |
| **Travel cost** |  |  |
| $cost_{drivers}$ | Car rental, driver wage, and gasoline | 90.00 |
