# Supplementary material for: An intuitive sampling framework for setting-specific decision-making in soil-transmitted helminthiasis control programs
Source: PLoS Negl Trop Dis. 2026 Jun 5;20(6):e0014026. doi: 10.1371/journal.pntd.0014026 (PMC13258144; doi:10.1371/journal.pntd.0014026)
Supplement: S3 Table — The most conservative κ corresponds to the one κ that yields the largest sample size. (DOCX) [file pntd.0014026.s008.docx]

**Table S3. Prior mean and corresponding most conservative degree of certainty when determining survey design to scale down or declare EPHP.** The most conservative *κ* corresponds to the one *κ* that yields the largest sample size**.**

| **Target** | **Prevalence threshold (%)** | **Prior mean (*µ*)** | **Most conservative *κ*** |
| --- | --- | --- | --- |
| Scaling down PC | 2% | 1% | 200 |
|  | 10% | 5% | 562 |
|  | 20% | 15% | 215 |
|  | 50% | 40% | 600 |
|  |  |  |  |
| Elimination as a public health problem | 2% | 1% | 200 |
