# Supplementary material for: An intuitive sampling framework for setting-specific decision-making in soil-transmitted helminthiasis control programs
Source: PLoS Negl Trop Dis. 2026 Jun 5;20(6):e0014026. doi: 10.1371/journal.pntd.0014026 (PMC13258144; doi:10.1371/journal.pntd.0014026)
Supplement: S4 Table — This table represents the required sample size (nschools ×nchildren), the decision cut-off c and the total survey cost (Ctot) for surveys based on screening one stool sample with a single Kato-Katz thick smear (KK1×1). Note that we assumed the maximum number of children per school. Also, we set the risk of undertreating to 1% while fixing the maximum allowed the risk of overtreatment was 20%, a certainty level κ of 200 and prior mean of 1%. (DOCX) [file pntd.0014026.s009.docx]

**Table S4. The cost-efficient survey design to declare EPHP.** This table represents the required sample size ($n_{schools} \times n_{children}$), the decision cut-off $c$ and the total survey cost ($C_{tot}$) for surveys based on screening one stool sample with a single Kato-Katz thick smear ($KK_{1\times1}$). Note that we assumed the maximum number of children per school. Also, we set the risk of undertreating to 1% while fixing the maximum allowed the risk of overtreatment was 20%, a certainty level of 200 and prior mean of 1%.

| **STH species** | **Survey design** | $\boldsymbol{n}_{\boldsymbol{schools}}$ | $\boldsymbol{n}_{\boldsymbol{children}}$ | $\boldsymbol{c}$ | $\boldsymbol{C}_{\boldsymbol{tot}}$ **(**$\boldsymbol{US\$)}$ |
| --- | --- | --- | --- | --- | --- |
| *Ascaris* | $KK_{1 \times1}$ | 9 | 62 | 8 | 4,322 |
| Hookworm | $KK_{1 \times1}$ | 11 | 74 | 11 | 5,539 |
| *Trichuris* | $KK_{1 \times1}$ | 10 | 42 | 6 | 4,414 |
|  |  |  |  |  |  |
| *Ascaris* | $KK_{1 \times2}$ | 9 | 62 | 8 | 4,400 |
| Hookworm | $KK_{1 \times2}$ | 11 | 74 | 11 | 5,959 |
| *Trichuris* | $KK_{1 \times2}$ | 10 | 48 | 7 | 4,598 |

$KK_{1\times2}$: duplicate Kato-Katz thick smear on a single stool sample; $KK_{1\times1}$: single Kato-Katz thick smear on a single stool sample; $n_{schools}$: number of schools; $n_{children}:$ required number of children per school; $c$: decision cut-off (maximum number of positive individuals that triggers a decision to continue PC at the same frequency); PC: preventive chemotherapy; EPHP: elimination as a public health problem.
