# Supplementary material for: An intuitive sampling framework for setting-specific decision-making in soil-transmitted helminthiasis control programs
Source: PLoS Negl Trop Dis. 2026 Jun 5;20(6):e0014026. doi: 10.1371/journal.pntd.0014026 (PMC13258144; doi:10.1371/journal.pntd.0014026)
Supplement: S5 Table — In this table, we fixed the maximum risk of undertreatment at 1% and overtreatment at 20% for switching to an event-based PC or scaling down PC frequency. To this end, we first identified the required sample size to switch to event-based PC when considering a prior mean of 1% and 1.4% and two options for the degree of certainty: high (200) and low (800). We further determined the corresponding decision for scaling down the PC frequency while using the required sample for 2% threshold and their corresponding prior mean. (DOCX) [file pntd.0014026.s010.docx]

**Table S5. Recommended survey designs for decision making in STH control programs.** In this table, we fixed the maximum risk of undertreatment at 1% and overtreatment at 20% for switching to an event-based PC or scaling down PC frequency. To this end, we first identified the required sample size to switch to event-based PC when considering a prior mean of 1% and 1.4% and two options for the degree of certainty: high (200) and low (800). We further determined the corresponding decision for scaling down the PC frequency while using the required sample for 2% threshold and their corresponding prior mean.

| **Threshold** | **Prior mean (%)** | **Certain** | **Survey design** | $\boldsymbol{n}_{\boldsymbol{schools}}$ | $\boldsymbol{n}_{\boldsymbol{children}}$ | **STH-specific decision cut-off** $\boldsymbol{c}$ | | |
| --- | --- | --- | --- | --- | --- | --- | --- | --- |
|  |  |  |  |  |  | Hookworm | *Ascaris* | *Trichuris* |
| **Switch to event-based PC (2%) or scale down the frequency of PC (10%, 20%, 50%)** | | | | | | | | |
| 2% | 1 | High | $KK_{1\times2}$ | 6 | 56 | 7 | 7 | 7 |
| 10% | 5 | High | $KK_{1\times2}$ | 6 | 56 | 27 | 26 | 27 |
| 20% | 15 | High | $KK_{1\times2}$ | 6 | 56 | 74 | 68 | 73 |
| 50% | 40 | High | $KK_{1\times2}$ | 6 | 56 | 178 | 170 | 178 |
|  |  |  |  |  |  |  |  |  |
| 2% | 1 | Low | $KK_{1\times2}$ | 3 | 30 | 3 | 3 | 3 |
| 10% | 5 | Low | $KK_{1\times2}$ | 3 | 30 | 9 | 8 | 9 |
| 20% | 15 | Low | $KK_{1\times2}$ | 3 | 30 | 22 | 20 | 22 |
| 50% | 40 | Low | $KK_{1\times2}$ | 3 | 30 | 52 | 49 | 51 |
|  |  |  |  |  |  |  |  |  |
| 2% | 1.4 | High | $KK_{1\times2}$ | 16 | 100 | 31 | 30 | 29 |
| 10% | 5 | High | $KK_{1\times2}$ | 16 | 100 | 118 | 110 | 117 |
| 20% | 15 | High | $KK_{1\times2}$ | 16 | 100 | 324 | 305 | 324 |
| 50% | 40 | High | $KK_{1\times2}$ | 16 | 100 | 801 | 767 | 809 |
|  |  |  |  |  |  |  |  |  |
| 2% | 1.4 | Low | $KK_{1\times2}$ | 10 | 68 | 14 | 13 | 13 |
| 10% | 5 | Low | $KK_{1\times2}$ | 10 | 68 | 52 | 49 | 52 |
| 20% | 15 | Low | $KK_{1\times2}$ | 10 | 68 | 142 | 133 | 142 |
| 50% | 40 | Low | $KK_{1\times2}$ | 10 | 68 | 349 | 333 | 350 |
